# Downregulation of Filamin a Expression in the Aorta Is Correlated With Aortic Dissection

**DOI:** 10.3389/fcvm.2021.690846

**Published:** 2021-08-13

**Authors:** Yue Chen, Xiang Wei, Zihao Zhang, Yi He, Bo Huo, Xian Guo, Xin Feng, Ze-Min Fang, Ding-Sheng Jiang, Xue-Hai Zhu

**Affiliations:** ^1^Division of Cardiothoracic and Vascular Surgery, Sino-Swiss Heart-Lung Transplantation Institute, Tongji Hospital, Tongji Medical College, Huazhong University of Science and Technology, Wuhan, China; ^2^Key Laboratory of Organ Transplantation, Ministry of Education, Chinese Academy of Medical Sciences, Wuhan, China; ^3^NHC Key Laboratory of Organ Transplantation, Chinese Academy of Medical Sciences, Wuhan, China; ^4^Key Laboratory of Organ Transplantation, Chinese Academy of Medical Sciences, Wuhan, China

**Keywords:** aortic dissection, FLNA, filamins, FOS/JUN, AP-1, bioinformatics

## Abstract

Filamins (FLNs) are actin cross-linking proteins, and as scaffolding proteins, FLNs are closely associated with the stabilization of the cytoskeleton. Nevertheless, the biological importance of FLNs in aortic dissection (AD) has not been well-elucidated. In this study, we first reanalyzed datasets downloaded from the Gene Expression Omnibus (GEO) database, and we found that in addition to the extracellular matrix, the actin cytoskeleton is a key structure associated with AD. Given that FLNs are involved in remodeling the cytoskeleton to affect cellular functions, we measured their expression levels in the aortas of patients with Stanford type A AD (TAAD). Our results showed that the mRNA and protein levels of FLNA were consistently decreased in dissected aortas of both humans and mice, while the FLNB protein level was upregulated despite decreased FLNB mRNA levels, and comparable expression levels of FLNC were observed between groups. Furthermore, the immunohistochemistry results demonstrated that FLNA was highly expressed in smooth muscle cells (SMCs) of aorta in non-AD samples, and downregulated in the medial layer of the dissected aortas of humans and mice. Moreover, we revealed that FOS and JUN, forming a dimeric transcription factor called AP-1 (activating protein-1), were positively correlated with the expression of FLNA in aorta. Either overexpression of FOS or JUN alone, or overexpression of FOS and JUN together, facilitated the expression of FLNA in primary cultured human aortic SMCs. In the present study, we not only detected the expression pattern of FLNs in aortas of humans and mice with or without AD, but we also found that the expression of FLNA in the AD samples was significantly reduced and that AP-1 might regulate the expression of FLNA. Our findings will contribute to the elucidation of the pathological mechanisms of AD and provide potential therapeutic targets for AD.

## Introduction

Aortic dissection (AD) is a life-threatening cardiovascular disorder requiring urgent surgical therapy. Despite advances in diagnostic modalities, surgical treatments, and medical devices, the mortality of AD remains high, and almost 75% of patients with Stanford type A AD (TAAD) die within 2 weeks when untreated (1). Hence, emergency surgery is currently the optimal solution for saving the lives of patients with TAAD. However, the outcome is unpredictable and patients may suffer many complications, such as spinal cord ischemia, stroke, mesenteric ischemia/infarction, and acute renal failure during rapid open surgical repair (2). Therefore, further exploration of the pivotal molecular mechanisms of AD to identify effective therapeutic targets is urgently needed.

Actins and actin-binding proteins are cytoskeletal proteins that are indispensable for cellular structure and function (3). In the aorta, these structural proteins are closely related to SMC contractile force and play a crucial role in vascular tension (4). Thus, the aberrant expression of these molecules can result in remodeling of the aorta and vascular disorder. For example, mutations in ACTA2, which encodes SMC α-actin, are responsible for 14% of patients with inherited ascending thoracic aortic aneurysms and dissections (5). ACTA2 deficiency in mice reduces elastin levels with increased collagen deposition, facilitating angiotensin II (Ang II)-induced TAAD (6). In addition to actins, smooth muscle 22 (SM22)-α, an SMC-specific actin-binding protein, is a contractile marker for SMC phenotype transition (7). The expression of SM22 is decreased in TAAD tissues and the suppression of SM22 in SMCs significantly contributes to proliferation, indicating the transformation from contractile to synthetic SMCs (7). Additionally, the depletion of actin-binding protein Girdin attenuates the proliferation and migration of SMCs to affect vascular remodeling (8). Thus, these studies indicated that actins and actin-binding proteins are vital for the stabilization of SMC functions and vascular homeostasis.

Filamins (FLNs) are actin-filament-crosslinking proteins and consist of three homologous proteins: FLNA, FLNB, and FLNC (9). As actin-binding proteins, FLNs can stabilize delicate three-dimensional actin webs and link them to cellular membranes to maintain cellular morphology (9). It was reported that FLNs interact with more than 70 proteins including transmembrane receptors and signaling molecules to play a vital role in cell motility, adhesion, spreading, and signal transduction (10). For SMCs, the degradation of FLNs inhibits differentiation and migration and interrupts phenotype switching (11). FLNA is the most abundant and widely understood actin-binding protein among the three FLN isoforms. Kevin Retailleau et al. reported that the deletion of FLNA in SMCs might cause a reduction in arterial stiffness and a compensatory increase in the conduit artery diameter of mice (12). Furthermore, FLNA is involved with a G protein-coupled P2Y2 nucleotide receptor to regulate the migration of vascular SMCs (13). However, the role of the FLN family in AD remains unclear.

In the present study, we first analyzed the differentially expressed genes in aortic samples of normal subjects and patients with AD, and further focused on the expression of the FLN family. Compared with non-AD samples, significantly reduced FLNA mRNA and protein levels were observed in the aortic samples obtained from AD patients. We next generated a murine AD model by treating mice with β-aminopropionitrile (BAPN) for 4 weeks and found a similar expression pattern of FLNA in normal and AD mice. Further bioinformatics analysis suggested that the expression levels of FOS and JUN, which form the activator protein-1 (AP-1) complex, were positively correlated with FLNA expression in the aorta of humans. Moreover, both FOS and JUN overexpression can promote the expression of FLNA in cultured primary human aorta smooth muscle cells (HASMCs). Thus, our data indicate that the AP-1/FLNA axis might play a potential role in AD formation and may serve as a promising therapeutic target for AD.

## Materials and Methods

### Human Aortic Samples

All protocols using human specimens were approved by the Human Research Ethics Committees of Tongji Hospital, Tongji Medical College, Huazhong University of Science and Technology, and informed consent was obtained from patients or their family members. Aortic tissues were obtained from patients with TAAD and patients undergoing heart transplantation (controls). All samples were stored in liquid nitrogen or paraformaldehyde as soon as possible after aorta excision to avoid specimen degradation.

### Animal Experiments

All animal experiments were performed in accordance with the protocols approved by the Animal Care and Use Committees of Tongji Hospital, Tongji Medical College, Huazhong University of Science and Technology. All mice (C57BL/6 background) were housed in specific-pathogen-free facilities with a 12-h light/dark cycle and controlled temperature (20–22°C). To induce AD, the 3-week-old mice were stimulated with 0.6% β-aminopropionitrile (BAPN, A3134; Sigma-Aldrich) taken orally for 4 weeks. The physical conditions of the mice were observed every day and autopsy was performed when the mice died.

### Western Blotting

Western blotting was performed as previously described (14). Human aorta specimens were homogenized with RIPA lysis buffer containing protease inhibitor complex and phosphatase inhibitors, and the protein concentration was assayed using a BCA protein assay kit (23227, Thermo Fisher Scientific). Twenty micrograms of protein were separated by 10% sodium dodecyl sulfate-polyacrylamide gel electrophoresis and then transferred to polyvinylidene fluoride membranes (IPVH00010, Millipore). The membranes were blocked with 5% nonfat milk and then incubated with primary antibodies at 4°C overnight. Subsequently, the membranes were washed and incubated with the corresponding horseradish peroxidase-conjugated secondary antibody. Finally, the membranes were incubated in ECL reagents prior to visualization using a ChemiDocTM XRS+ system (Bio-Rad). The antibodies used in this study included FLNA (ab76289, Abcam), FLNB (GTX101206, GeneTex), FLNC (ab180941, Abcam), HA (H3663, Sigma), Flag (F1084, Sigma), and β-Actin (AC026, ABclonal).

### Real-Time PCR

Real-time PCR was performed using established protocols (15). Total RNA from human aorta specimens was extracted using TRIzol reagent (15596026, Ambion) according to the manufacturer's instructions. In total, 5 μg of RNA was reverse-transcribed using the Transcriptor First Strand cDNA synthesis kit. The relative mRNA levels of FLN family members were detected by a quantitative real-time PCR system using SYBR green (11201ES08, Yeasen), and the results were normalized against 18S expression. Primer sequences are as follows: FLNA forward primer 5′-CCGCAATGACAATGACACC-3′, FLNA reverse primer 5′-TGGAGATACTGCCACTGAGA-3′, FLNB forward primer 5′-ACTGTCATGGCCACAGATGG-3′, FLNB reverse primer 5′-AAATCCCAGGCCGTTCATGT-3′, FLNC forward primer 5′- CTCCAGCTACAGCTCCATCC-3′, FLNC reverse primer 5′-CCATGTGCTTCACGTACACC-3′, and 18S forward primer 5′-CTCAACACGGGAAACCTCAC-3′, 18S reverse primer 5′-CGCTCCACCAACTAAGAACG-3′.

### Histology and Immunohistochemistry Staining

Histology and immunohistochemistry staining were performed as previously described (16). Human and mouse aortic samples were fixed in 4% paraformaldehyde and embedded in paraffin, and then 5-μm-thick serial sections were stained with hematoxylin-eosin (H&E) for morphological examination. Elastin fibers were visualized using elastic van Gieson (EVG) staining according to the manufacturer's instructions and quantified by counting the total breaks over the length of the vessel. Immunohistochemistry analyses were performed using a standard protocol. The paraffin-embedded sections were incubated with citrate antigen retrieval solution (pH 6.0, P0083, Beyotime) for antigen retrieval. The sections were incubated with primary antibodies overnight at 4°C and then incubated with horseradish peroxidase (HRP)-conjugated secondary antibodies for 1 h. Finally, the DAB horseradish peroxidase color development kit (ZLI-9017, ZSGB-BIO) was used for color development.

### Cell Culture

Primary HASMCs were isolated from the aortas of patients who underwent heart transplantation as previously described (14). Briefly, aortas were stored in DME/F12 medium (SH30023.01; HyClone) at 4°C, and then the intima and adventitia were stripped under microscope. Furthermore, the medial layer was peeled as thin as possible and minced in a sterile culture flask. Small pieces were placed in the wall of a culture flask without culture medium for 30 min to enable their adherence to the flask. Next, DME/F12 medium containing 10% fetal bovine serum (FBS, SH30084.03; HyClone) and 1% penicillin-streptomycin (15140-122; Thermo Fisher Scientific) was slowly added to the culture flask, which was maintained in a humidified environment at 37°C with 5% CO_2_. A week later, HASMCs were removed from tissues and then transferred to new culture dishes as first generation. After two passages of growth, the third generation SMCs were used for the cell experiments.

### Plasmid Constructs and Cell Treatments

The full-length coding sequences of the human FOS and JUN genes were amplified from cDNA and then cloned into the pHAGE vector as pHAGE-FOS-HA and pHAGE-JUN-Flag, respectively. pHAGE-FOS-HA and pHAGE-JUN-Flag plasmids, and the packaging plasmids psPAX2 (12260, Addgene) and pMD2.G (12259, Addgene) were cotransfected into HEK293T cells in the presence of polyethylenimine (764604, Sigma-Aldrich) and incubated for 48 h and the supernatants containing lentivirus were harvested and filtered through a 0.22 μm filter (SLGP033RB, Millipore) for cell infection. HASMCs were infected with the corresponding lentivirus (control, lenti-FOS-HA, lenti-JUN-Flag, lenti-FOS-HA+ lenti-JUN-Flag) for 24 h via hexadimethrine bromide (10 μg/mL, H9268, Sigma-Aldrich) and then cultured with DMEM/F12 medium without FBS for cell synchronization. Finally, the infected cells were maintained in DMEM/F12 medium containing FBS for 24 h and then collected for subsequent research.

### Bioinformatic Analysis

To obtain the mRNA expression profiles of aortas with or without AD in humans, the keywords “AD” and “Homo sapiens” were applied to search datasets in the Gene Expression Omnibus (GEO) database (https://www.ncbi.nlm.nih.gov/geo/). In these results, we further analyzed each dataset and excluded all non-mRNA datasets, such as non-coding RNA and DNA methylation profile sets. After assessing all the datasets independently, we included four gene expression datasets “GSE153434,” “GSE98770,” “GSE52093,” and “GSE147026,” which are datasets of expression profile array or mRNA high-throughput sequencing of aorta with or without AD in humans. To analyze the data, the raw expression values of each dataset were normalized by using the “limma” package in R, and log2 conversion was completed. After processing, differentially expressed genes (DEGs) were filtered based on the criteria adjust.p.value (FDR) <0.05 and logFC >1.5. The DEGs obtained in each dataset were further analyzed by the R package “clusterProfiler” for GO, KEGG, and GSE analysis (17). Each term was represented as a circle node, in which color represented its adjusted *p*-value. In addition, the node sizes indicated gene ratios and the mean proportions of genes enriched in the GO or KEGG categories.

To explore the transcription factors regulating FLNA, we queried the database of the website signaling pathways project (https://www.signalingpathways.org/index.jsf) and combined the results with the GSE153434 database to analyze the correlation between the expression of transcription factors and FLNA. The Spearman correlation coefficient was used in the correlation analysis, and *t*-tests were carried out to determine the significant differences.

### Statistical Analysis

The data are presented as the mean ± standard deviation (SD). All statistical analyses in this study were performed by using SPSS software (version 23.0). Comparisons of the means between 2 groups were performed by independent sample *t*-test and one-way ANOVA was performed for the difference assessments of more than two groups. Correlation analyses were performed using Pearson's correlation analysis. A value of *p* <0.05 was considered statistically significant.

## Results

### Bioinformatics Analysis of mRNA Expression Profiles in Human Aortas

**Figure 1:**
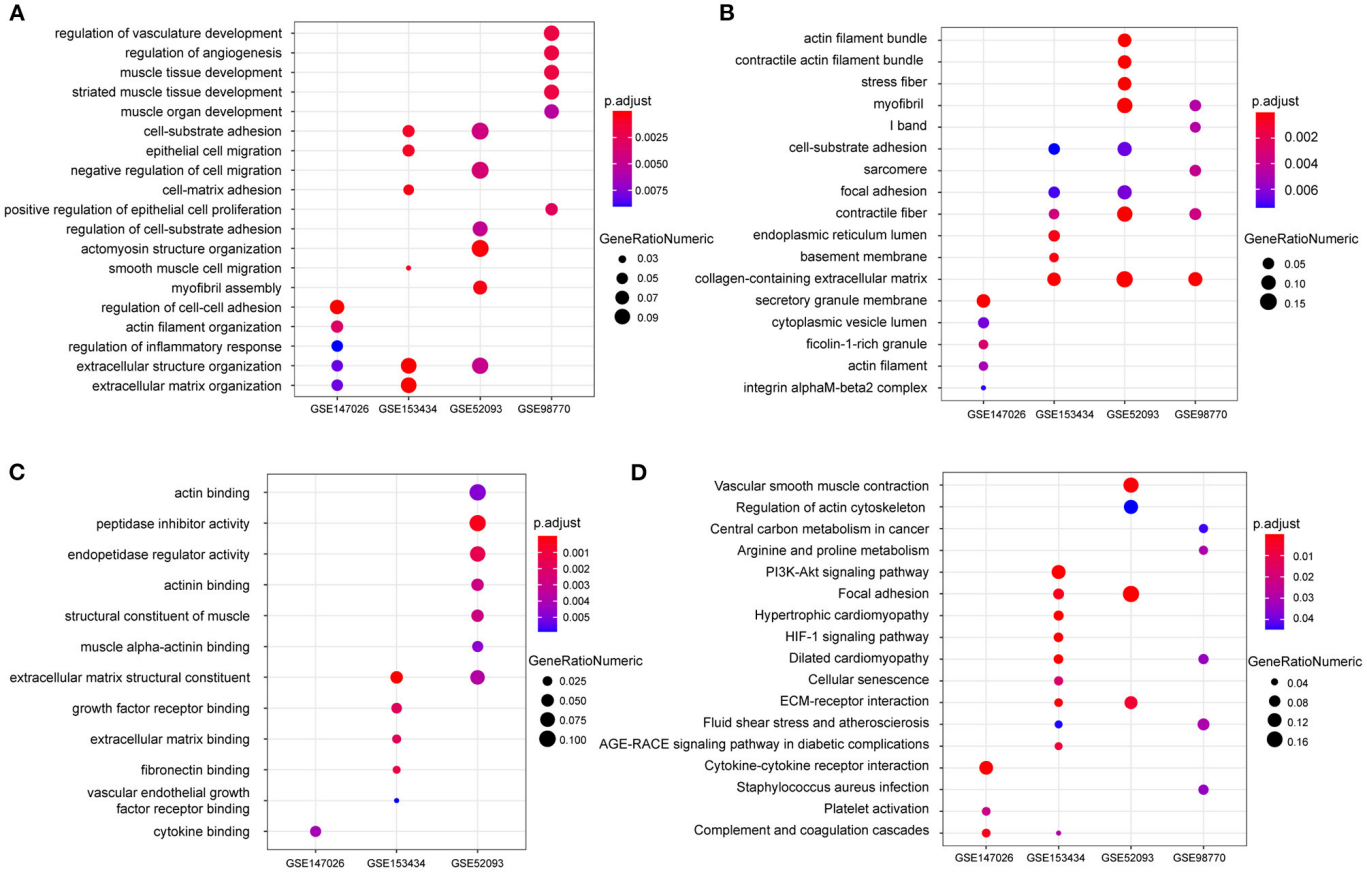
The enrichment analysis shows the differentially expressed genes between the AD and normal groups. **(A–C)** GO analysis showing the differences of mRNA expression in the aorta between AD and normal groups on biological processes **(A)** cellular component **(B)** or molecular function **(C)** among the four GEO datasets GSE147026, GSE153434, GSE52093, and GSE98770. **(D)** KEGG enrichment analysis showing the dramatically altered pathways in AD among the indicated datasets.

**Figure 2:**
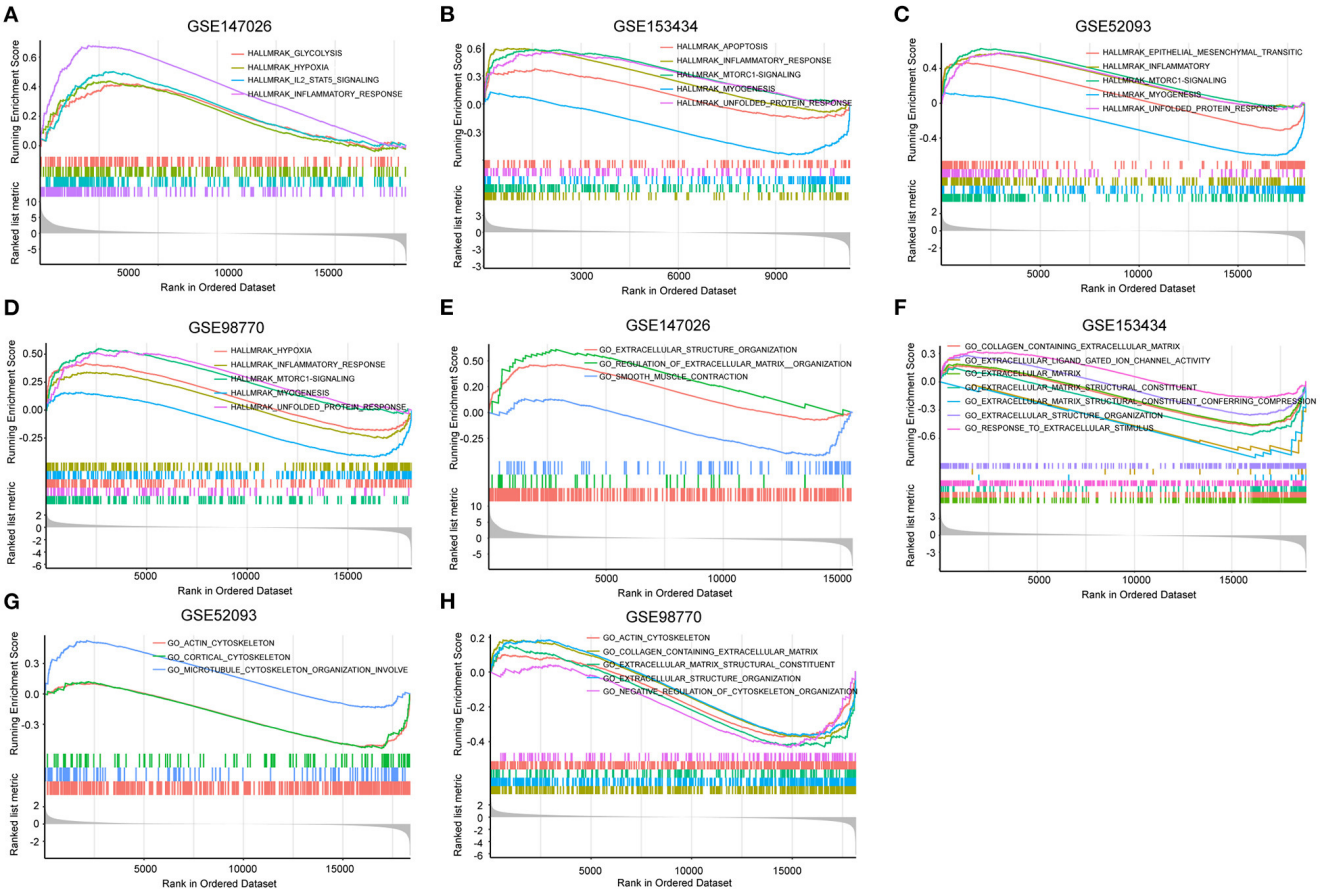
Gene set enrichment analysis (GSEA) was performed to identify gene networks associated with AD. **(A–D)** Visualization of remarkable enrichment hallmark terms among the four GEO datasets GSE147026, GSE153434, GSE52093, and GSE98770. **(E–H)** Visualization of remarkablely enriched C5 collection terms among the four GEO datasets GSE147026, GSE153434, GSE52093, and GSE98770.

To explore the differential gene expression in normal controls and patients with TAAD, we reanalyzed four gene expression datasets—GSE147026, GSE153434, GSE52093, and GSE98770—downloaded from the GEO database. In terms of biological process, extracellular structure organization was enriched in these four datasets (Figure 1A). Similarly, cellular component and molecular function analysis revealed that extracellular matrix (ECM) was the most highly enriched GO term (Figures 1B,C), consistent with previous investigations (18). In addition to the ECM, actin associated elements were also key factors used to elucidate the pathological process of AD (Figures 1A–C), such as actin filaments and actin-binding proteins. Furthermore, the KEGG pathway enrichment analysis displayed conspicuous differences in actin cytoskeleton pathways between control and TAAD samples (Figure 1D). Notably, the inflammatory response, which participates in SMC death and ECM degradation, was identified as a common TAAD associated cause in the four datasets according to gene set enrichment analysis (GSEA) (Figures 2A–D). Moreover, the actin cytoskeleton was enriched again by using the C5 collection (ontology gene sets) used for the GSEA (Figures 2E–H). Thus, these results indicated that abnormalities in the actin cytoskeleton may be involved in the development of TAAD in humans.

### The mRNA Expression of FLNA and FLNB Is Downregulated in Patients With TAAD

**Figure 3:**
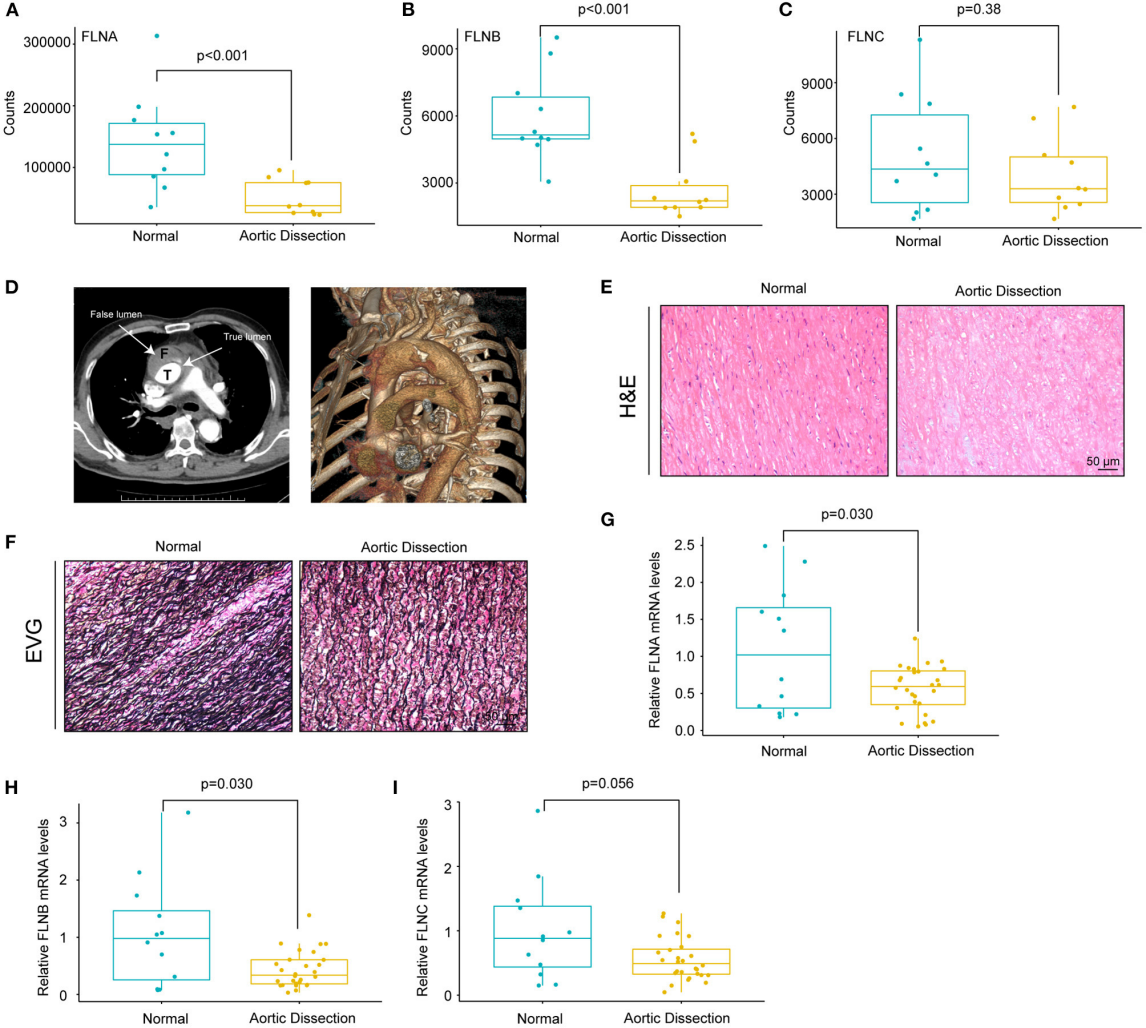
The mRNA levels of FLNA and FLNB were downregulated in human TAAD tissues. **(A–C)** The expression of FLNA **(A)** FLNB **(B)** and FLNC **(C)** in non-AD and dissected human aortas based on GSE153434 dataset. **(D)** Representative iterative reconstruction CTA images of TAAD patients, T, true lumen; F, false lumen. **(E,F)** Representative images of H&E-stained **(E)** and EVG-stained **(F)** aortic sections from non-AD and dissected human aortas. **(G–I)** The relative mRNA levels of FLNA **(G)** FLNB **(H)** and FLNC **(I)** in the aortas of humans from normal donors (*n* = 14) and AD (*n* = 33) patients.

Given that the actin cytoskeleton was enriched and because FLN family members are actin-binding proteins, we hypothesized that FLN family members may be involved in the pathogenesis of AD. Therefore, we first analyzed the expression levels of FLNs in the GSE153434 dataset. The results showed that FLNA and FLNB expression was decreased in the aorta of the AD patients, whereas FLNC displayed no difference between the two groups (Figures 3A–C). To validate the results of the dataset analysis, we collected aortic tissues from TAAD patients and non-AD aortas from patients who underwent heart transplantation. In this research, all enrolled TAAD patients were diagnosed through CT angiography (CTA), and true/false lumens were clearly visible in the CTA images (Figure 3D). Significant destructive ECM remodeling of the ascending aorta in AD identified according to its morphology (Figure 3E). Compared with normal aorta tissues, TAAD aorta tissues showed disorganized elastin fibers with increased fragmentation as indicated by elastin van Gieson (EVG) staining (Figure 3F). We further detected the mRNA levels of FLNs in the aorta of non-AD and TAAD patients by real-time PCR and found lower mRNA levels of FLNA and FLNB in patients with TAAD than in control subjects (Figures 3G,H). However, comparable mRNA levels of FLNC were observed between the two groups (Figure 3I).

**Figure 4:**
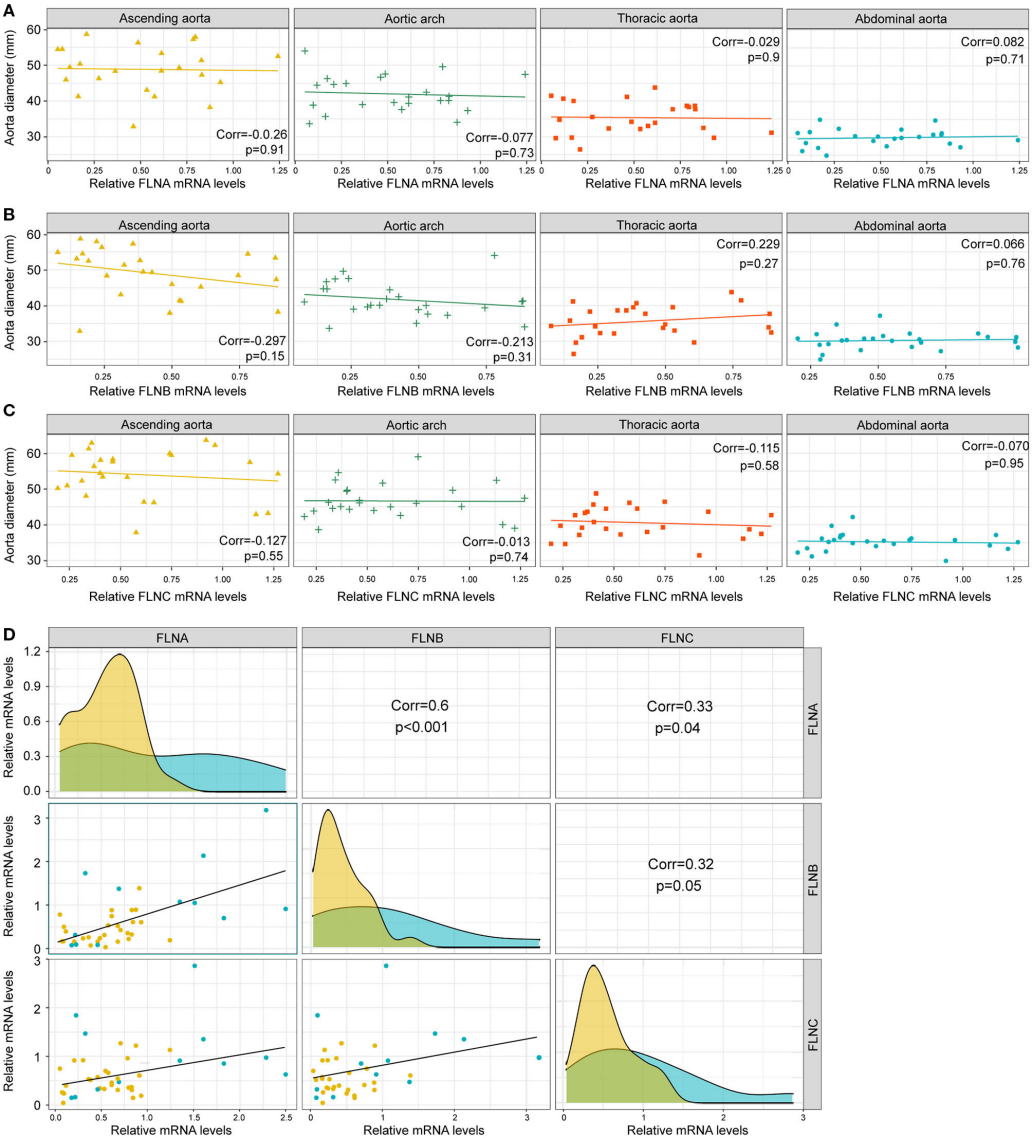
The expression of FLNA and FLNB had a significantly positive correlation. **(A–C)** Correlation analysis showing the relationship between FLNA **(A)** FLNB **(B)** and FLNC **(C)** expression and the diameters of ascending, thoracic and abdominal aorta, as well as the aortic arch. **(D)** Correlation analysis showing the internal connection of mRNA levels among FLNA, FLNB, and FLNC.

Since dilatation of the aorta is one of the characteristics of AD patients, we next investigated the relationship between the mRNA expression of FLNs and aortic diameters. As shown in Figures 4A–C, there was no remarkable correlation between the FLN mRNA levels and the diameters of aorta, including the ascending, the thoracic and abdominal aorta, or aortic arch. In contrast, the mRNA expression of FLNA and FLNB was positively correlated in the aorta (*r* = 0.6, *p* < 0.001), but only a weak correlation was found between FLNA and FLNC and between FLNB and FLNC (Figure 4D). Thus, considering the similar expression level of FLNC between the non-AD and AD samples, FLNC might not be involved in the process of AD and should not be further explored in future research. Together, these results suggested that FLNA and FLNB might be involved in the development of AD.

### The Protein Levels of FLNA and FLNB in TAAD Patients

**Figure 5:**
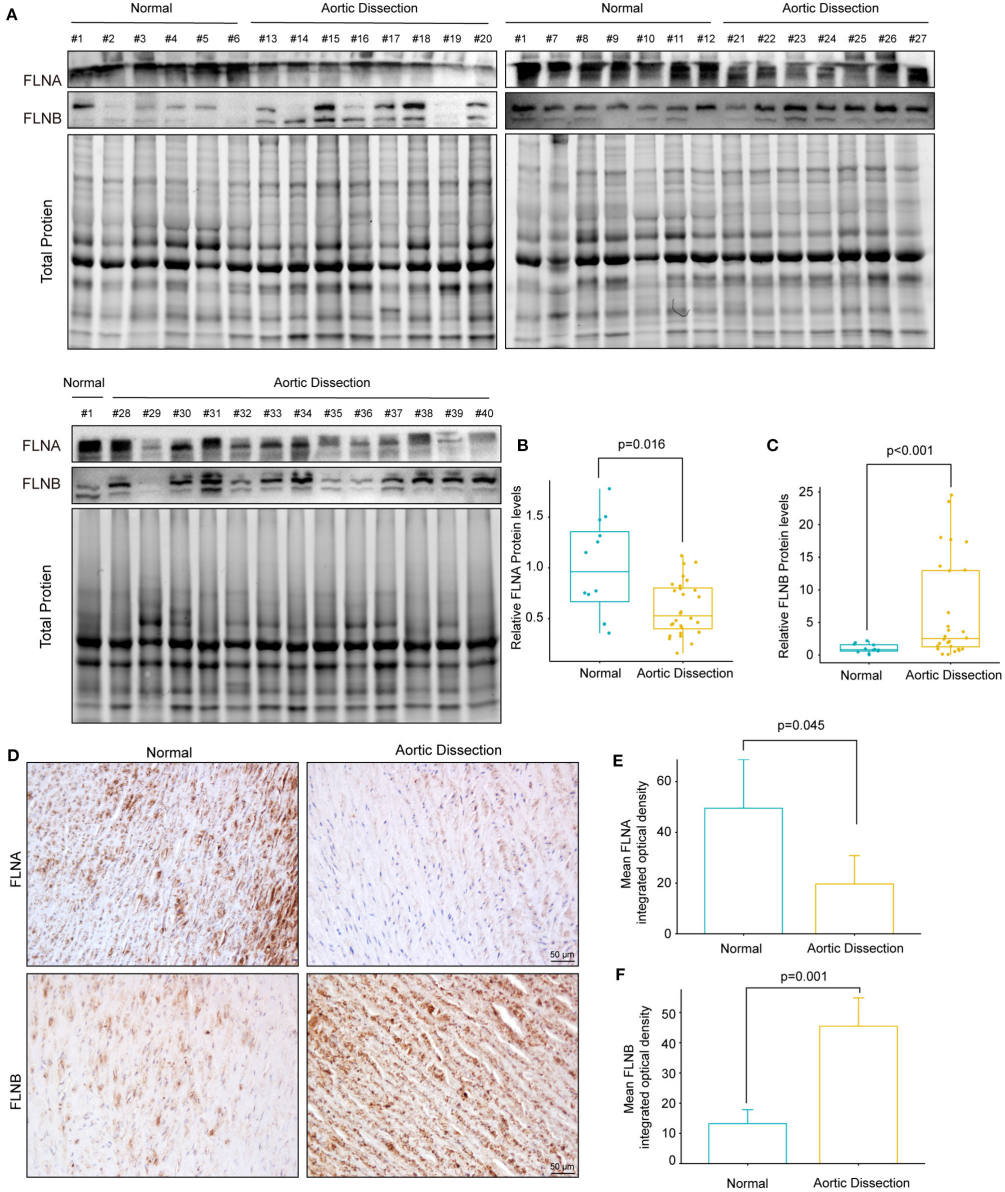
The protein expression of FLNA and FLNB was completely opposite in human TAAD tissues. **(A–C)** Western blotting and quantitative results of FLNA and FLNB in aortic samples obtained from normal donors (*n* = 12) and AD (*n* = 28) patients. **(D–F)** Representative images of immunohistochemical staining and quantitative results of FLNA and FLNB in the aortic sections of normal and AD patients (*n* = 3).

We further investigated whether the protein level of FLNA was altered during the process of AD. Total protein was extracted from aorta with or without TAAD, and the protein levels of FLNA and FLNB were detected by Western blotting. The results showed that consistent with changes in mRNA levels, the protein level of FLNA also obviously decreased in the TAAD samples compared with that in the controls (Figures 5A,B). Similarly, the results of immunohistochemistry showed that FLNA was highly expressed in the normal aorta but significantly reduced in the aorta of patients with TAAD (Figures 5D,E). However, in contrast to the decrease in mRNA level, the protein level of FLNB significantly increased in the aorta of patients with TAAD, and the results of immunohistochemistry further verified this result (Figures 5A,C,D,F). Thus, these results further indicated that FLNA is a candidate gene affecting AD development.

### The Mouse AD Model Was Successfully Established With Young Mice Through the Administration of BAPN

**Figure 6:**
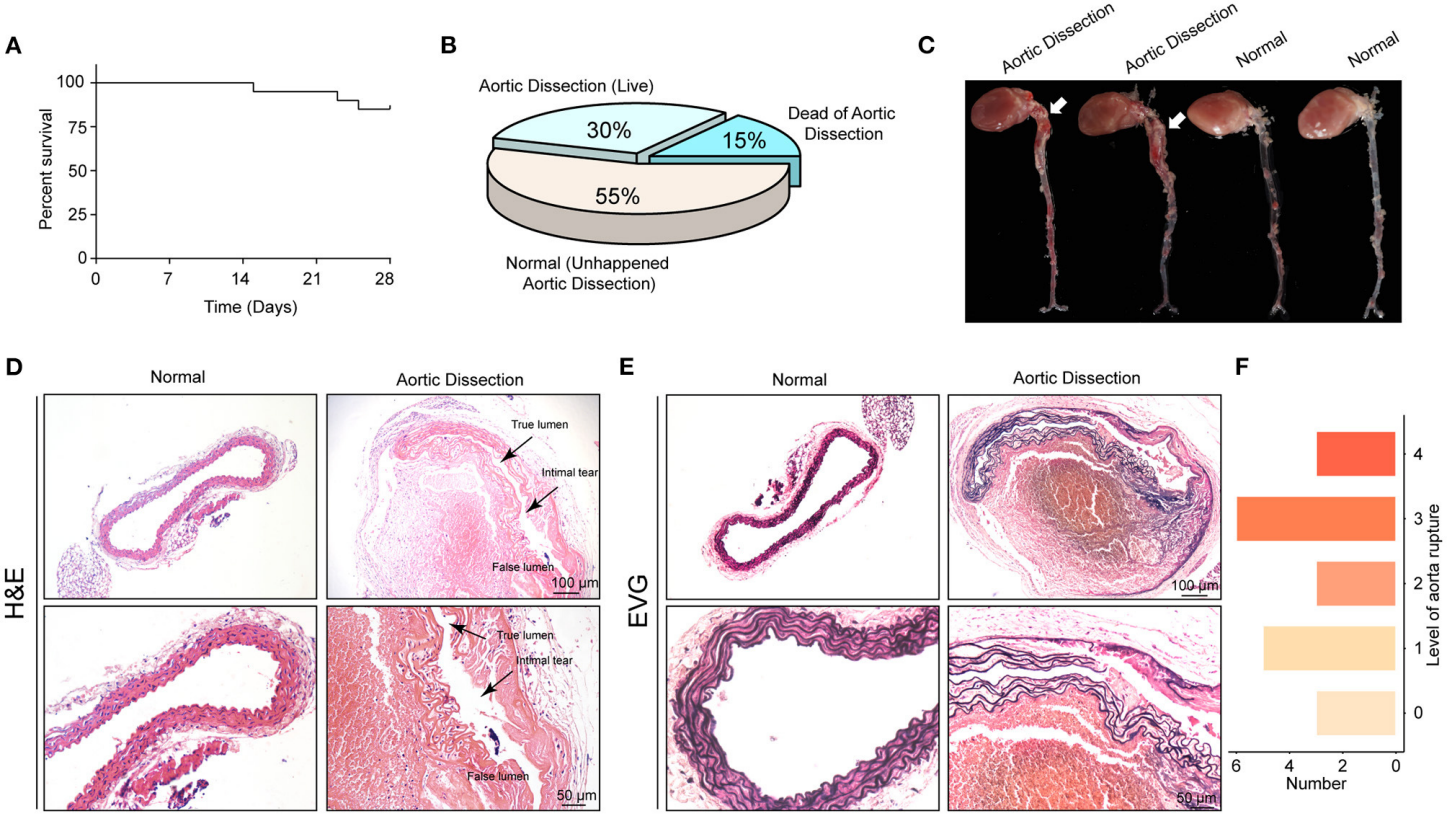
The AD model was successfully established with young mice by administering BAPN. **(A)** Survival curves of the male mice treated with 0.6% β-aminopropionitrile (BAPN) when 3 weeks old. **(B)** The overall incidences of indicated conditions in mice after 4 weeks of BAPN treatment. **(C)** Representative macroscopic images of excised aortas after 4 weeks of BAPN treatment. **(D,E)** Representative images of H&E-stained **(D)** and EVG-stained **(E)** aorta sections from non-AD and dissected mouse aortae. The true/false lumens and intimal tears were labeled in the dissected aorta. **(F)** Distribution of the degree of elastin rupture in mice treated with BAPN treatment. *n* = 20.

To examine the roles of the FLN family *in vivo*, BAPN was used to generate AD models in young mice (19). First, we treated 3-week-old male mice with 0.6% BAPN *per os* for 4 weeks and monitored the condition of the mice daily. We found that after 2 weeks of BAPN treatment, the mice began to die because of dissection rupture. The mortality of BAPN-treated mice after 4 weeks was 15% (Figure 6A) and the morbidity of AD was 45% according to macroscopic autopsy (Figures 6B,C). Dissection mainly occurred in the ascending aorta, aorta arch, and thoracic aorta (Figure 6C). Furthermore, the aorta of the dissected mice had pathological changes similar to those of the human aorta, including destructive ECM remodeling and disruption of elastic fibers (Figures 6D,E). Consistently, H&E staining showed an intimal tear between the true and false lumens, and displayed inflammatory cell infiltration in the false lumen (Figure 6D). According to the degree of elastic fiber fragmentation in the aorta (0, 0–25%, 25–50%, 50–75%, and 75–100%), we divided the medial degeneration of the aorta into 5 levels (0, 1, 2, 3, and 4) (20). After 4 weeks of BAPN treatment, aortas of 3 mice were almost normal, and aortas of the remaining mice exhibited elastic fiber breakage to varying degrees (Figure 6F). In conclusion, we successfully constructed a mouse AD model that can simulate the occurrence of human AD by stimulating young mice with BAPN.

### FLNA Was Downregulated in the Aortas of Mice Treated With BAPN

**Figure 7:**
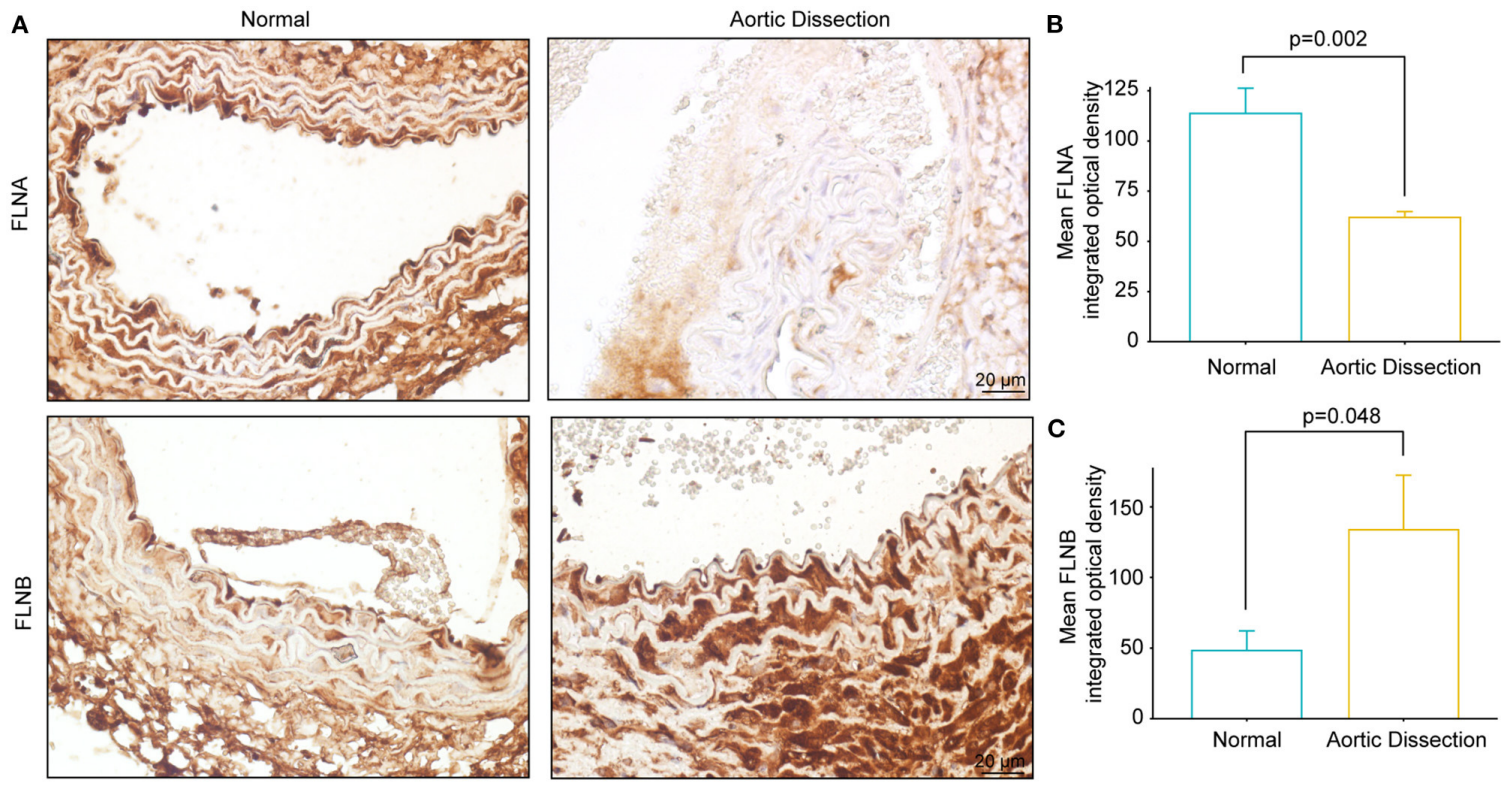
The expression of FLNA and FLNB in mice with AD is similar to that in human tissue. **(A–C)** Representative images of immunohistochemical staining and quantitative results of FLNA and FLNB in the aorta sections of the normal and BAPN-induced AD mice (*n* = 3).

Using a mouse model of AD, we further examined the FLNA and FLNB protein levels via immunohistochemistry. Consistent with our observations in human samples, FLNA was highly expressed in normal aortae, while FLNB was expressed at low levels (Figure 7A). Furthermore, the protein level of FLNA in the aorta of BAPN-treated mice was significantly decreased compared with its expression in controls (Figures 7A,B), while an obvious increase in FLNB was detected in the aorta of mice after treatment with BAPN (Figures 7A,C). Collectively, the expression patterns of FLNA and FLNB in human AD tissue were confirmed in the mice, which suggested that they might be indispensable for the pathogenesis of AD.

### AP-1 Facilitates FLNA Expression in HASMCs

**Figure 8:**
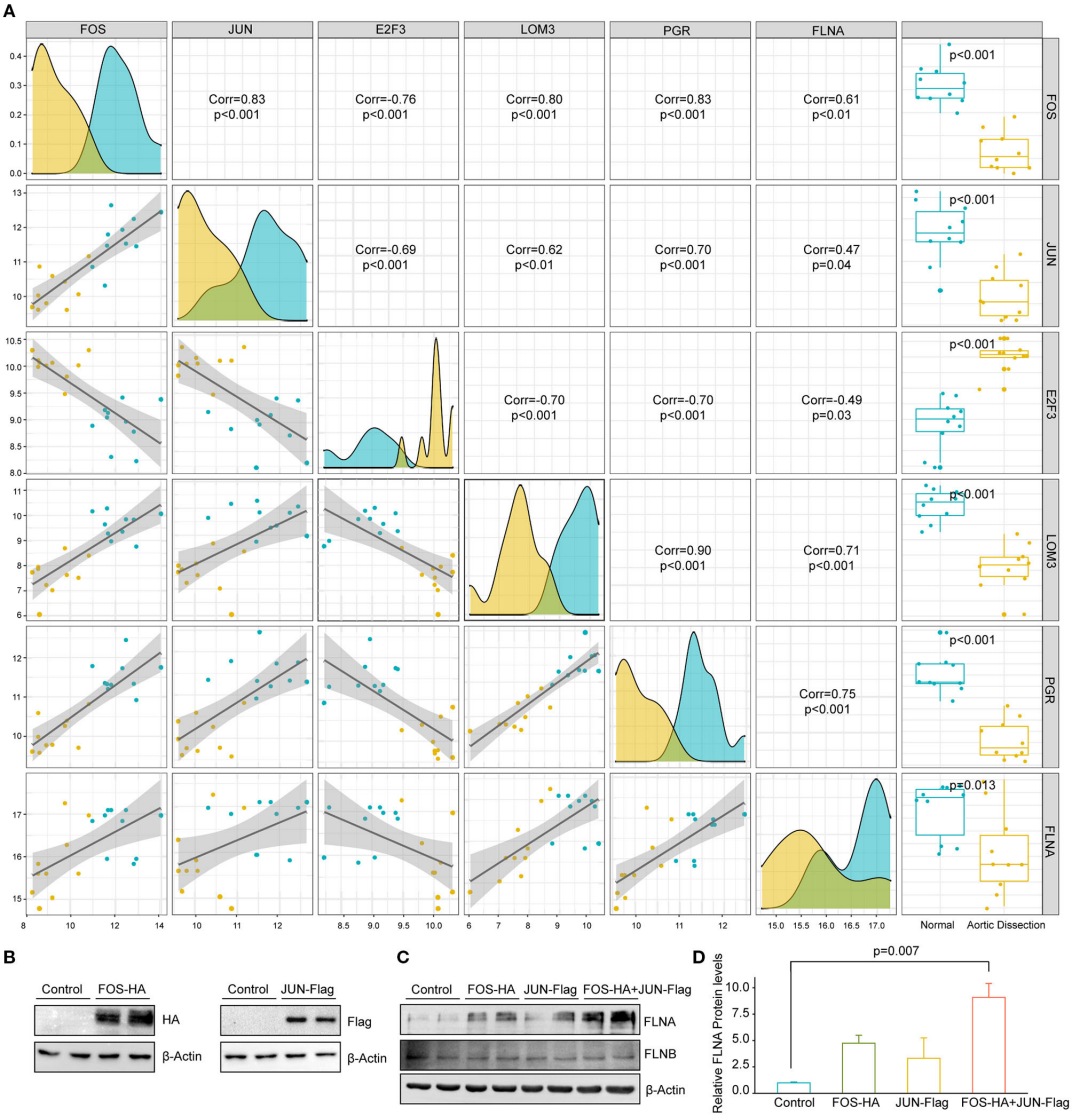
FOS/JUN contributes to FLNA expression in HASMCs. **(A)** The five transcription factors mostly related to FLNA transcriptional regulation. **(B)** Western blotting of FOS and JUN expression in HASMCs infected with lenti-FOS-HA and lenti-JUN-Flag. **(C,D)** Western blot and quantitative results showing FLNA and FLNB expression in HASMCs infected with corresponding lentivirus (Control, FOS-HA, JUN-Flag, FOS-HA+JUN-Flag). β-Actin served as the loading control.

To further explore the upstream mechanism that inhibited FLNA expression during TAAD development, we collected transcription factors that were reported to be able to bind to the FLNA promoter, as indicated by chromatin immunoprecipitation assay-sequence (ChIP-seq) data obtained from The Signaling Pathways Project (https://www.signalingpathways.org). We then screened these transcription factors in the GSE153434 dataset to identify differentially expressed transcription factors that potentially regulate FLNA expression. Our screening results showed that five transcription factors, FOS, JUN, E2F3, LMO3, and PGR, were differentially expressed in the GSE153434 dataset. Among these factors, FOS, JUN, LOM3, and PGR was downregulated in patients with AD and positively correlated with FLNA, while E2F3 expression was upregulated (Figure 8A). Notably, FOS and JUN are both subunits of the transcription factor AP-1, which is essential for the function of VSMCs and associated with AD (21). Therefore, we were very interested to know whether AP-1 is a transcription factor that regulates FLNA expression. Therefore, we overexpressed FOS and JUN in HASMCs via lentivirus infection (Figure 8B). The results showed that either FOS or JUN overexpression in HASMCs promoted FLNA expression, which was enhanced to a greater extent by simultaneous overexpression of FOS and JUN (Figures 8C,D). However, the expression of FLNB was not regulated by FOS or JUN, as evidenced by the comparable expression level of FLNB between the indicated groups of HASMCs (Figure 8C). These results indicated that AP-1 might be the transcription factor regulating FLNA expression during AD occurrence.

## Discussion

In this study, by reanalyzing datasets in the GEO database, we identified the actin cytoskeleton as a key component in AD development. We further investigated the expression pattern of the FLN family during AD and found that the mRNA and protein expression levels of FLNA were significantly downregulated in the aortic wall of AD patients compared with those in the control aorta. Furthermore, a similar scenario was observed in the mouse AD model. Thus, it is highly plausible that FLNA participates in the process of AD. Finally, we found that the transcription complex FOS/JUN can promote the expression of FLNA, which indicated the regulatory mechanism of FLNA in AD.

FLNs are important actin crosslinking proteins and their biological function is largely limited to cell migration (9). Heike Roth et al. showed that FLNA can facilitate efficient migration of human neutrophil-like HL-60 cells via reducing activation of myosin-II (22). It was reported that neutrophils egress from marrow and infiltrate into the aortic adventitia in BAPN/Ang II-treated mice and then release matrix metalloproteinases, such as MMP8 and MMP9, resulting in the degradation of the ECM and the progression of AD (23–25). In addition, Ningpu Yu et al. showed that P2Y2R regulated the proliferation and migration of vascular SMCs by binding FLNA (13). It is well-known that SMCs exhibit two different phenotypes, acting as contractile or synthetic SMCs, and cells with the contractile phenotype, the predominant form in normal aorta, exhibit relatively low migration and proliferation capacity and high contractile capability (26). Several previous studies confirmed that phenotypic transformation of SMCs from the contractile to synthetic type is one of the typical events in the development of AD (27, 28). The increase of migration ability is the embodiment of the synthetic type, accompanied by the secretion of matrix metalloproteinases and a decline in contractile capability (29, 30). Thus, downregulated FLNA expression in AD patients may be involved in the regulation of migration ability, phenotype transformation, and ECM secretion by VSMCs.

Other studies found that some functions of FLNA in addition to migration might be associated with AD. In the patients with Ehlers-Danlos syndrome and periventricular heterotopia, caused by a loss-of-function mutation in the FLNA gene, was detected and found to contribute the development of aortic dilatation in early adulthood (31). Indeed, the C-terminal fragment of FLNA was previously proposed as a new biomarker of arterial wall remodeling in hypertension (32), suggesting that FLNA is of crucial importance for vascular structure. Feng et al. demonstrated that FLNA is necessary for cell junctions in vascular development, and FLNA-null mice displayed failure of vascular remodeling, accompanied by coarse and dilated blood vessels (33). Moreover, smooth muscle-specific FLNA knockout in mice only at the adult stage led to prominent vascular abnormalities, including a reduction in arterial stiffness and a compensatory increase in conduit artery diameter (12). In AD, arterial stiffness is increased owing to the deposition of collagen, resulting in the enhanced aorta susceptibility to dissection and rupture (34). Consistent with aortic dilation as one of the most important risk factors for AD, our results in the present study showed that FLNA is decreased in the aortas of AD patients. These findings indicated that reduced FLNA may contribute to aortic dilation and remodeling during AD. Further studies are needed to verify this hypothesis, especially using knockout or overexpression approaches in animal models.

Similar to FLNA, FLNB also participates in the regulation of migration in many cells. FLNB mediates the firm adhesion of leukocytes to the endothelium through interaction with ICAM-1(35), resulting in the transendothelial migration of leukocytes. The recruitment of leukocytes to the vessel endothelium results in a local inflammatory response, tears in the intima, and the initiation of AD. In addition, S Bandaru et al. showed that FLNB deficiency enhanced the activity of MMP9 and secretion of vascular endothelial growth factor (VEGF)-A via the RAS/ERK pathway, resulting in tumor growth and metastasis (36). The roles of MMP9 and VEGF have been exhaustively studied in patients with AD. MMP9 degrades type IV collagen, elastin, and various basement membrane proteins of SMCs (26), while VEGF contributes to the proinflammatory actions and neoangiogenesis process in aortic wall remodeling (37). It is surprising that the mRNA level of FLNB was decreased, whereas the protein expression increased. The inconsistency between the mRNA and protein levels of FLNB might be attributed to the following causes. First, mRNA posttranscriptional modification is a familiar form of mRNA stability and translation regulation, such as N6-methyladenosine (M^6^A) modification (38). For example, ADARB1, an adenosine-to-inosine (A-to-I) RNA-editing enzyme, mediates circadian rhythms through mRNA, and plays a role in the posttranscriptional regulation of FLNB (39). In addition, the ubiquitin-proteasome degradation pathway was verified as another mechanism for FLNB post-translational regulation. Heuze et al. reported that ASB2 ubiquitin ligase activity drives proteasome-mediated degradation of the actin-binding protein FLNB and then inhibits cell spreading on fibronectin (40). The E3 ubiquitin ligase specificity subunit ASB2α regulates cell spreading, migration, and differentiation by interacting with the filamin actin-binding domain, which induces FLNB proteasomal degradation (41). Moreover, a novel ASB2 isoform, ASB2β, targets FLNB for proteasomal degradation and impacts myoblast fusion and myotube formation (42). Hence, the ubiquitin proteasome system is a vital pathway for FLNB degradation, and the upregulated FLNB protein level might be attributed to insufficient of degradation in the process of AD, which should be further investigated.

At the mRNA level, we showed that the expression of both FLNA and FLNB was downregulated and that a strong positive correlation between FLNA and FLNB expression was observed in the aortic wall of AD tissues. This result indicates the possibility that FLNA and FLNB have similar transcriptional regulation mechanisms. Filamin family members have a similar structure and show 70% amino acid sequence homology (43), suggesting potential functional compensation between FLNA and FLNB. A yeast two-hybrid experiment demonstrated that the FLNB homodimerization domain can strongly interact with the corresponding homologous region of FLNA, and then, via immunoprecipitation assays, FLNA-FLNB heterodimers were verified to exist (44). Previous studies have also demonstrated that FLNA and FLNB play overlapping roles in stabilizing the actin cytoskeleton and cell function (45). In terms of migration, loss of FLNA or FLNB has no effect on migration, but it impairs the initiation of cell migration (46). In chondrocytes, it was demonstrated that loss of FLNA induces upregulation of FLNB expression, and vice versa (47). Taken together with the opposite changes in the protein levels of FLNA and FLNB in AD, it is possible that FLNB upregulation may compensate for the decrease in FLNA. Of course, different and even opposite functions have also been illuminated in other areas of study. For instance, in RAS-induced lung tumorigenesis, knockout of FLNA significantly reduced the tumor formation and proliferation of fibroblasts via inactivation of ERK and AKT (48). Nevertheless, in contrast to FLNA, FLNB deficiency enhanced RAS-induced tumor growth and metastasis through the RAS/ERK pathway (49).

Another important finding reported here is the transcriptional regulation function of the AP-1 complex (FOS/JUN) on FLNA. To date, few studies have reported the regulatory mechanism of FLNA. Matthew R. Sarkisian et al. revealed that MEKK4 suppression contributes to abnormally high FLNA expression and inhibits neuronal migration (50). In the present study, we revealed that AP-1 might promote FLNA expression in HASMCs. AP-1 is a member of the JUN, FOS, Maf, and ATF subfamilies (51) and well-recognized as a key transcription factor in cell proliferation, death, and oncogenesis (52). It was reported that IL-18 facilitated AP-1-dependent MMP9 transcription, resulting in increased SMC migration ability (53). Investigation into the possible regulation of FLNA by AP-1 suggested that FLNA might be critical for AP-1-mediated SMC migration. Moreover, AP-1 is also a pivotal factor for AD and regulates medial degeneration (54). Zhang et al. reported that the protein expression of AP-1 in the aorta of AD was downregulated compared to that in normal aortas, and SIRT1 activated AP-1/decorin signaling to alleviate AD (21). Neutralization of AP-1 via decoy oligodeoxynucleotides repressed aortic elastolysis with reduced fiber breaks and MMP activity in a Marfan syndrome mouse model (54). Therefore, we suspect that the inhibition of AP-1 in SMCs aggravates the development of AD, and we suggest that more investigations are directed to the AP-1/FLNA axis in AD.

In addition to AD, aortic aneurysm is also a vessel disease caused by the medial degeneration. However, there are many differences between dissection and aneurysm. Indeed, there might be aorta dilation before AD formation, but it would not progress to aneurysm. The rupture of aneurysms is limited, whereas the rupture of AD shows the formation of true/false lumens, accompanied by extension of the tears. In addition, the most common locations of AD are the ascending aorta, aorta arch, and thoracic aorta (55), while aneurysms mainly occur on the abdominal aorta (56). Of course, the formation of AD is accompanied by dilation of the aorta, and aneurysms would develop into dissection and rupture of the aorta. However, it must be emphasized that dissection and aneurysm are two different diseases, not two forms of one disease, which should be distinguished in both clinical treatment and basic research. Currently, CT angiography has widely been applied to AD diagnosis, and intimal tears and true/false lumens can be clearly distinguished (57).

There are some limitations in this study. First, we detected the FLN family expression in the aortas of mice and patients diagnosed with AD, but functions of FLNs have not been investigated, especially using knockout or overexpression approach in animal models. Second, only the BAPN induced mouse AD model was used to analyze FLNs expression. Two or more mouse models of AD would make this conclusion more convincing and meaningful. Finally, we found the possibility of AP-1 on FLNA transcriptional regulation via bioinformatics and further validated that AP-1 could promote FLNA expression in HASMCs. However, further experiments should be done to strengthen this regulation mechanism and to illustrate their functions in AD development.

In summary, our study demonstrated that the expression level of FLNA was reduced in the aortas of patients with AD, and AP-1 might be the transcription factor mediating the expression of FLNA in HASMCs. These results indicated that the AP-1/FLNA axis may be essential for the occurrence of AD, and targeting the AP-1/FLNA axis may be a novel therapeutic strategy for AD.

## Data Availability Statement

The datasets presented in this study can be found in online repositories. The names of the repository/repositories and accession number(s) can be found in the article/supplementary material.

## Ethics Statement

The studies involving human participants were reviewed and approved by the Human Research Ethics Committees of Tongji Hospital, Tongji Medical College, Huazhong University of Science and Technology. The patients/participants provided their written informed consent to participate in this study. The animal study was reviewed and approved by the Animal Care and Use Committees of Tongji Hospital, Tongji Medical College, Huazhong University of Science and Technology.

## Author Contributions

YC and XW performed the cell and animal experiments of this study and data analysis. ZZ and YH participated in bioinformatics analysis. BH and XG cultured the primary HASMCs and constructed the plasmid. XF and Z-MF collected the human aorta tissues. X-HZ and D-SJ designed the work and finished the manuscript. All authors contributed to the article and approved the submitted version.

## Conflict of Interest

The authors declare that the research was conducted in the absence of any commercial or financial relationships that could be construed as a potential conflict of interest.

## Publisher's Note

All claims expressed in this article are solely those of the authors and do not necessarily represent those of their affiliated organizations, or those of the publisher, the editors and the reviewers. Any product that may be evaluated in this article, or claim that may be made by its manufacturer, is not guaranteed or endorsed by the publisher.
